# Regenerative medicine in the treatment of specific dermatologic disorders: a systematic review of randomized controlled clinical trials

**DOI:** 10.1186/s13287-024-03800-6

**Published:** 2024-06-18

**Authors:** Alireza Jafarzadeh, Arash Pour Mohammad, Haniyeh Keramati, Roya Zeinali, Mina Khosravi, Azadeh Goodarzi

**Affiliations:** 1 Department of Dermatology, Rasool Akram Medical Complex Clinical Research Development Center (RCRDC), School of Medicine, Rasool Akram Hospital, Iran University of Medical Sciences (IUMS), Niayesh Street, Sattar Khan Avenue, Tehran, 1445613131 Iran; 2 Faculty of Medicine, Iran University of Medical Sciences, Shahid Hemmat Highway, Tehran, 1449614535 Iran

**Keywords:** Regenerative medicine, Dermatological disorder, Review, Systematic review, PRP, SVF, Androgenetic alopecia, AGA, Alopecia areata, Vitiligo, Melasma, LSA, Telogen effluvium, Psoriasis, Conditioned medium, Mesenchymal stem cells, Autologous epidermal melanocyte/keratinocyte cells, Dystrophic epidermolysis bullosa, Concentrated growth factor, Acne, Lichen planus, Erosive oral lichen planus, Basic fibroblast growth factor, Platelet-rich plasma, Stromal vascular fraction

## Abstract

**Aims and objectives:**

The aim of this study is to systematically review randomized controlled clinical trials (RCTs) studying various types of regenerative medicine methods (such as platelet-rich plasma, stromal vascular fraction, cell therapy, conditioned media, etc.) in treating specific dermatologic diseases. Rejuvenation, scarring, wound healing, and other secondary conditions of skin damage were not investigated in this study.

**Method:**

Major databases, including PubMed, Scopus, and Web of Science, were meticulously searched for RCTs up to January 2024, focusing on regenerative medicine interventions for specific dermatologic disorders (such as androgenetic alopecia, vitiligo, alopecia areata, etc.). Key data extracted encompassed participant characteristics and sample sizes, types of regenerative therapy, treatment efficacy, and adverse events.

**Results:**

In this systematic review, 64 studies involving a total of 2888 patients were examined. Women constituted 44.8% of the study population, while men made up 55.2% of the participants, with an average age of 27.64 years. The most frequently studied skin diseases were androgenetic alopecia (AGA) (45.3%) and vitiligo (31.2%). The most common regenerative methods investigated for these diseases were PRP and the transplantation of autologous epidermal melanocyte/keratinocyte cells, respectively. Studies reported up to 68.4% improvement in AGA and up to 71% improvement in vitiligo. Other diseases included in the review were alopecia areata, melasma, lichen sclerosus et atrophicus (LSA), inflammatory acne vulgaris, chronic telogen effluvium, erosive oral lichen planus, and dystrophic epidermolysis bullosa. Regenerative medicine was found to be an effective treatment option in all of these studies, along with other methods. The regenerative medicine techniques investigated in this study comprised the transplantation of autologous epidermal melanocyte/keratinocyte cells, isolated melanocyte transplantation, cell transplantation from hair follicle origins, melanocyte–keratinocyte suspension in PRP, conditioned media injection, a combination of PRP and basic fibroblast growth factor, intravenous injection of mesenchymal stem cells, concentrated growth factor, stromal vascular fraction (SVF), a combination of PRP and SVF, and preserving hair grafts in PRP.

**Conclusion:**

Regenerative medicine holds promise as a treatment for specific dermatologic disorders. To validate our findings, it is recommended to conduct numerous clinical trials focusing on various skin conditions. In our study, we did not explore secondary skin lesions like scars or ulcers. Therefore, assessing the effectiveness of this treatment method for addressing these conditions would necessitate a separate study.

## What is already known about this topic?

- Regenerative medicine is an effective treatment method for managing inflammatory and degenerative diseases, including inflammatory skin diseases.
- Regenerative medicine methods encompass therapeutic approaches such as PRP injection, SVF injection, use of conditioned medium, autologous melanocyte and keratinocyte cell transplantation, exosomes, utilization of mesenchymal stem cells, and a combination of the aforementioned methods.
- So far, most studies on the role of regenerative medicine in dermatology have focused on rejuvenation, scar healing, and wound healing. However, there has been no systematic review of specific skin diseases.

## What does this study add?

- To date, clinical trial studies in the field of regenerative medicine for the treatment of specific dermatologic diseases such as androgenetic alopecia (AGA), vitiligo, alopecia areata, melasma, lichen sclerosus et atrophicus (LSA), inflammatory acne vulgaris, chronic telogen effluvium, erosive oral lichen planus, and dystrophic epidermolysis bullosa have been conducted.
- In relation to AGA, 86.2% of the studies have shown the effectiveness of various regenerative medicine methods in treating the disease. This rate is 100% for vitiligo, 83.3% for alopecia areata, 100% for melasma, 50% for LSA, 100% for acne vulgaris, 100% for chronic telogen effluvium, 100% for erosive oral lichen planus, and 100% for dystrophic epidermolysis bullosa.
- The most common diseases associated with the use of regenerative medicine in their treatment include androgenetic alopecia and vitiligo. The most common methods in AGA include PRP, Conditioned Media, and a Combination of PRP and Basic Fibroblast Growth Factor (bFGF), respectively. Regenerative medicine has been effective in treating AGA up to 68.4% in studies.
- In the case of vitiligo, the most common methods are Transplantation of autologous epidermal melanocyte/keratinocyte cells, isolated melanocyte transplantation, and Cell transplantation from hair follicle origins, with the treatment showing effectiveness up to 71% in studies for this disease.

## Introduction

Regenerative medicine is a crucial field of medicine known for its effectiveness in treating numerous inflammatory and degenerative diseases. This treatment approach encompasses various methods such as platelet-rich plasma (PRP), stromal vascular fraction (SVF), conditioned media, and the transplantation of autologous epidermal melanocyte/keratinocyte cells [1–3].

These methods, through multiple pathways, ultimately lead to a singular result, which is the reversal of the inflammatory and degeneration processes by releasing cell factors and cytokines as well as promoting the proliferation of stem cells. For instance, the PRP injection method aims to release factors from concentrated platelets, whereas in the SVF injection, stem cells and factors derived from adipocyte cells stimulate cell regeneration [4, 5].

These treatments have been extensively investigated for a wide range of skin diseases, which can be categorized into two main groups: primary skin diseases, including androgenetic alopecia [6, 7], vitiligo [8, 9], alopecia areata [10], telogen effluvium [11], melasma [12], acne vulgaris [13], lichen sclerosus et atrophicus (LSA) [14], erosive oral lichen planus [15, 16], and dystrophic epidermolysis bullosa [17]; and skin conditions secondary to damage such as acne scars, hypertrophic scars from burns, erosions, and ulcers [18].

In our study, we conducted a systematic review of all regenerative medicine treatment methods for primary skin diseases. Specifically, we focused on randomized controlled clinical trials to assess recovery rates, side effects, and intervention protocols. We meticulously analyzed each treatment method to provide a comprehensive summary of their efficacy in managing various skin conditions.

## Method and materials

### Search strategy and databases

This systematic review was conducted in accordance with the Preferred Reporting Items for Systematic Reviews and Meta-Analyses (PRISMA) guidelines. A meticulous literature review was performed to identify relevant keywords for different types of regenerative medicine, including fat transfer, stem cell therapy, SVF, PRP, platelet-poor plasma, and melanocyte–keratinocyte transplantation, among others. The dermatological conditions encompassed Lichen Planopilaris, Frontal Fibrosing Alopecia, Androgenetic Alopecia, other forms of Alopecia (Areata, Telogen Effluvium, Anagen Effluvium), Cicatricial and Non-Cicatricial Alopecia, Discoid Lupus Erythematosus, Psoriasis, Hidradenitis Suppurativa, Morphea, LSA, and also a broader category of dermatological diseases including Granulomatous Disease, Vasculitis, Inflammatory Skin and Cutaneous Diseases, Pyoderma Gangrenosum, Inflammatory Dermatoses, Genodermatoses, Adverse Drug Reactions, Toxic Epidermal Necrolysis, Stevens-Johnson Syndrome, Papulosquamous Disorders, Vitiligo, Pigmentary Disorders, Immunobullous Diseases (Pemphigus Vulgaris, Bullous Pemphigoid), Epidermolysis Bullosa, melanocytic and non-melanocytic Skin Cancers (Basal Cell Carcinoma, Squamous Cell Carcinoma), Ulcerative, Anogenital, and Nail Disorders, Collagen Vascular Disease, Acne, and Melasma. To refine the specificity of retrieved studies, keywords pertaining to randomized clinical trials were also integrated. Furthermore, a meticulous examination of the references within selected articles was conducted to encompass all relevant research. The search spanned studies published up to January 3, 2024. The detailed syntaxes utilized across different databases are delineated in Table 1.

**Table 1:** Search syntax across databases

PubMed	(“Adipose derived stem cells conditioned medium” OR “fat transfer” OR “nano fat” OR “lipo transfer” OR “fat grafting” OR “stem cell therapy” OR “stromal vascular fraction” OR “Extracellular vesicle” OR “ADSC” OR “platelet rich plasma” OR “stem cell” OR “lipografting” OR “melanocyte–keratinocyte transplantation” OR “epidermal melanocyte transplantation” OR “Autologous melanocyte–keratinocyte suspension” OR “Platelet-poor plasma” OR “lipofiling” OR “adipose tissue regeneration”) AND (“LPP” OR “lichen planopilaris” OR “FFA” OR “frontal fibrosing alopecia” OR “AGA” OR “Androgenetic alopecia” OR “Androgenic alopecia” OR “alopecia” OR “alopecia Areata” OR “telogen effluvium” OR “Anagen effluvium” OR “cicatricial alopecia” OR “non-cicatricial alopecia” OR “discoid lupus erythematosus” OR “DLE” OR “psoriasis” OR “lichen planus” OR “hidradenitis suppurativa” OR “morphea” OR “LSA” OR “Lichen sclerosus et atrophicus” OR “Lichen sclerosus” OR “dermatological disease” OR “granulomatous disease” OR “vasculitis” OR “inflammatory skin disease” OR “inflammatory cutaneous disease” OR “pyoderma gangrenosum” OR “inflammatory dermatoses” OR “genodermatoses” OR “drug reaction” OR “Toxic epidermal necrolysis” OR “Stevens-Johnson syndrome” OR “TEN/SJS” OR “papulosquamous disorders” OR “vitiligo” OR “pigmentary disorders” OR “immunobullous disease” OR “pemphigus vulgaris” OR “bullous pemphigoid” OR “epidermolysis bullosa” OR “melanocytic cancer” OR “BCC” OR “basal cell carcinoma” OR “SCC” OR “Squamous Cell Carcinoma” OR “ulcerative disease” OR “anogenital disease” OR “nail disorder” OR “non melanocytic skin cancer” OR “collagen vascular disease” OR “acne” OR “melisma”)
Scopus	( TITLE-ABS ( “randomized controlled trial”) OR TITLE ( “RCT”) OR TITLE ( “clinical trial”) OR TITLE ( “randomized”) OR TITLE ( “placebo-controlled”) OR TITLE ( “randomized controlled trial”) OR TITLE ( “randomly”) OR TITLE ( “double-blind”)) AND ( ( TITLE-ABS-KEY ( “LPP”) OR TITLE-ABS-KEY ( “lichen planopilaris”) OR TITLE-ABS-KEY ( “FFA”) OR TITLE-ABS-KEY ( “frontal fibrosing alopecia”) OR TITLE-ABS-KEY ( “AGA”) OR TITLE-ABS-KEY ( “Androgenetic alopecia”) OR TITLE-ABS-KEY ( “Androgenic alopecia”) OR TITLE-ABS-KEY ( “alopecia”) OR TITLE-ABS-KEY ( “alopecia Areata”) OR TITLE-ABS-KEY ( “telogen effluvium”) OR TITLE-ABS-KEY ( “Anagen effluvium”) OR TITLE-ABS-KEY ( “cicatricial alopecia”) OR TITLE-ABS-KEY ( “non-cicatricial alopecia”) OR TITLE-ABS-KEY ( “discoid lupus erythematosus”) OR TITLE-ABS-KEY ( “DLE”) OR TITLE-ABS-KEY ( “psoriasis”) OR TITLE-ABS-KEY ( “lichen planus”) OR TITLE-ABS-KEY ( “hidradenitis suppurativa”) OR TITLE-ABS-KEY ( “morphea”) OR TITLE-ABS-KEY ( “LSA”) OR TITLE-ABS-KEY ( “Lichen sclerosus et atrophicus”) OR TITLE-ABS-KEY ( “Lichen sclerosus”) OR TITLE-ABS-KEY ( “dermatological disease”) OR TITLE-ABS-KEY ( “granulomatous disease”) OR TITLE-ABS-KEY ( “vasculitis”) OR TITLE-ABS-KEY ( “inflammatory skin disease”) OR TITLE-ABS-KEY ( “inflammatory cutaneous disease”) OR TITLE-ABS-KEY ( “pyoderma gangrenosum”) OR TITLE-ABS-KEY ( “inflammatory dermatoses”) OR TITLE-ABS-KEY ( “genodermatoses”) OR TITLE-ABS-KEY ( “drug reaction”) OR TITLE-ABS-KEY ( “Toxic epidermal necrolysis”) OR TITLE-ABS-KEY ( “Stevens-Johnson syndrome”) OR TITLE-ABS-KEY ( “TEN/SJS”) OR TITLE-ABS-KEY ( “papulosquamous disorders”) OR TITLE-ABS-KEY ( “vitiligo”) OR TITLE-ABS-KEY ( “pigmentary disorders”) OR TITLE-ABS-KEY ( “immunobullous disease”) OR TITLE-ABS-KEY ( “pemphigus vulgaris”) OR TITLE-ABS-KEY ( “bullous pemphigoid”) OR TITLE-ABS-KEY ( “epidermolysis bullosa”) OR TITLE-ABS-KEY ( “melanocytic cancer”) OR TITLE-ABS-KEY ( “BCC”) OR TITLE-ABS-KEY ( “basal cell carcinoma”) OR TITLE-ABS-KEY ( “SCC”) OR TITLE-ABS-KEY ( “Squamous Cell Carcinoma”) OR TITLE-ABS-KEY ( “ulcerative disease”) OR TITLE-ABS-KEY ( “anogenital disease”) OR TITLE-ABS-KEY ( “nail disorder”) OR TITLE-ABS-KEY ( “non melanocytic skin cancer”) OR TITLE-ABS-KEY ( “collagen vascular disease”) OR TITLE-ABS-KEY ( “acne”) OR TITLE-ABS-KEY ( “melisma”)) AND ( ( TITLE-ABS-KEY ( “adipose derived stem cells conditioned medium”) OR TITLE-ABS-KEY ( “fat transfer”) OR TITLE-ABS-KEY ( “nano fat”) OR TITLE-ABS-KEY ( “lipo transfer”) OR TITLE-ABS-KEY ( “fat grafting”) OR TITLE-ABS-KEY ( “stem cell therapy”) OR TITLE-ABS-KEY ( “stromal vascular fraction”) OR TITLE-ABS-KEY ( “Extracellular vesicle”) OR TITLE-ABS-KEY ( “ADSC”) OR TITLE-ABS-KEY ( “platelet rich plasma”) OR TITLE-ABS-KEY ( “stem cell”) OR TITLE-ABS-KEY ( “lipografting”) OR TITLE-ABS-KEY ( “melanocyte–keratinocyte transplantation”) OR TITLE-ABS-KEY ( “epidermal melanocyte transplantation”) OR TITLE-ABS-KEY ( “Autologous melanocyte–keratinocyte suspension”) OR TITLE-ABS-KEY ( “Platelet-poor plasma”) OR TITLE-ABS-KEY ( “lipofiling”) OR TITLE-ABS-KEY ( “adipose tissue regeneration”)))) AND ( EXCLUDE ( DOCTYPE, “re”))
Web of science	TS = (“adipose derived stem cells conditioned medium” OR “fat transfer” OR “nano fat” OR “lipo transfer” OR “fat grafting” OR “stem cell therapy” OR “stromal vascular fraction” OR “Extracellular vesicle” OR “ADSC” OR “platelet rich plasma” OR “stem cell” OR “lipografting” OR “melanocyte–keratinocyte transplantation” OR “epidermal melanocyte transplantation” OR “Autologous melanocyte–keratinocyte suspension” OR “Platelet-poor plasma” OR “lipofiling” OR “adipose tissue regeneration”) AND TS = (“LPP” OR “lichen planopilaris” OR “FFA” OR “frontal fibrosing alopecia” OR “AGA” OR “Androgenetic alopecia” OR “Androgenic alopecia” OR “alopecia” OR “alopecia Areata” OR “telogen effluvium” OR “Anagen effluvium” OR “cicatricial alopecia” OR “non-cicatricial alopecia” OR “discoid lupus erythematosus” OR “DLE” OR “psoriasis” OR “lichen planus” OR “hidradenitis suppurativa” OR “morphea” OR “LSA” OR “Lichen sclerosus et atrophicus” OR “Lichen sclerosus” OR “dermatological disease” OR “granulomatous disease” OR “vasculitis” OR “inflammatory skin disease” OR “inflammatory cutaneous disease” OR “pyoderma gangrenosum” OR “inflammatory dermatoses” OR “genodermatoses” OR “drug reaction” OR “Toxic epidermal necrolysis” OR “Stevens-Johnson syndrome” OR “TEN/SJS” OR “papulosquamous disorders” OR “vitiligo” OR “pigmentary disorders” OR “immunobullous disease” OR “pemphigus vulgaris” OR “bullous pemphigoid” OR “epidermolysis bullosa” OR “melanocytic cancer” OR “BCC” OR “basal cell carcinoma” OR “SCC” OR “Squamous Cell Carcinoma” OR “ulcerative disease” OR “anogenital disease” OR “nail disorder” OR “non melanocytic skin cancer” OR “collagen vascular disease” OR “acne” OR “melisma”) AND TI = (“randomized controlled trial” OR “RCT” OR “clinical trial” OR “randomized” OR “placebo-controlled” OR “randomly” OR “double-blind”)

### Inclusion and exclusion criteria

The inclusion criteria were specifically designed to include RCTs that investigate the effectiveness of regenerative medicine interventions for treating dermatological disorders. The exclusion criteria targeted studies that deviated from the RCT design, such as non-randomized trials, review articles, studies focusing on secondary skin lesions (e.g., scars, ulcers), and interventions not categorized under regenerative medicine (e.g., topical treatments, traditional pharmacotherapy). Additionally, non-human research (including animal models and in vitro studies), experimental studies, and trials lacking definitive outcome measures were excluded. Articles not written in English or those without accessible full texts were also omitted.

### Study selection and data extraction

Two evaluators [A.J. and H.K] reviewed the titles and abstracts of identified records based on the predefined inclusion criteria. They assessed each study for various attributes, including the study design, the dermatological condition being assessed, the type of regenerative therapy, the treatment regimen, observed improvements, and reported adverse events. Details such as average age, gender distribution, and the number of participants were also collected from these studies. To facilitate the article screening and data extraction processes, EndNote® X8 and Google Sheets™ were employed. This screening and data compilation were carried out independently by two investigators, with any discrepancies being adjudicated by consulting a senior researcher [A.G.].

## Result

**Fig. 1:**
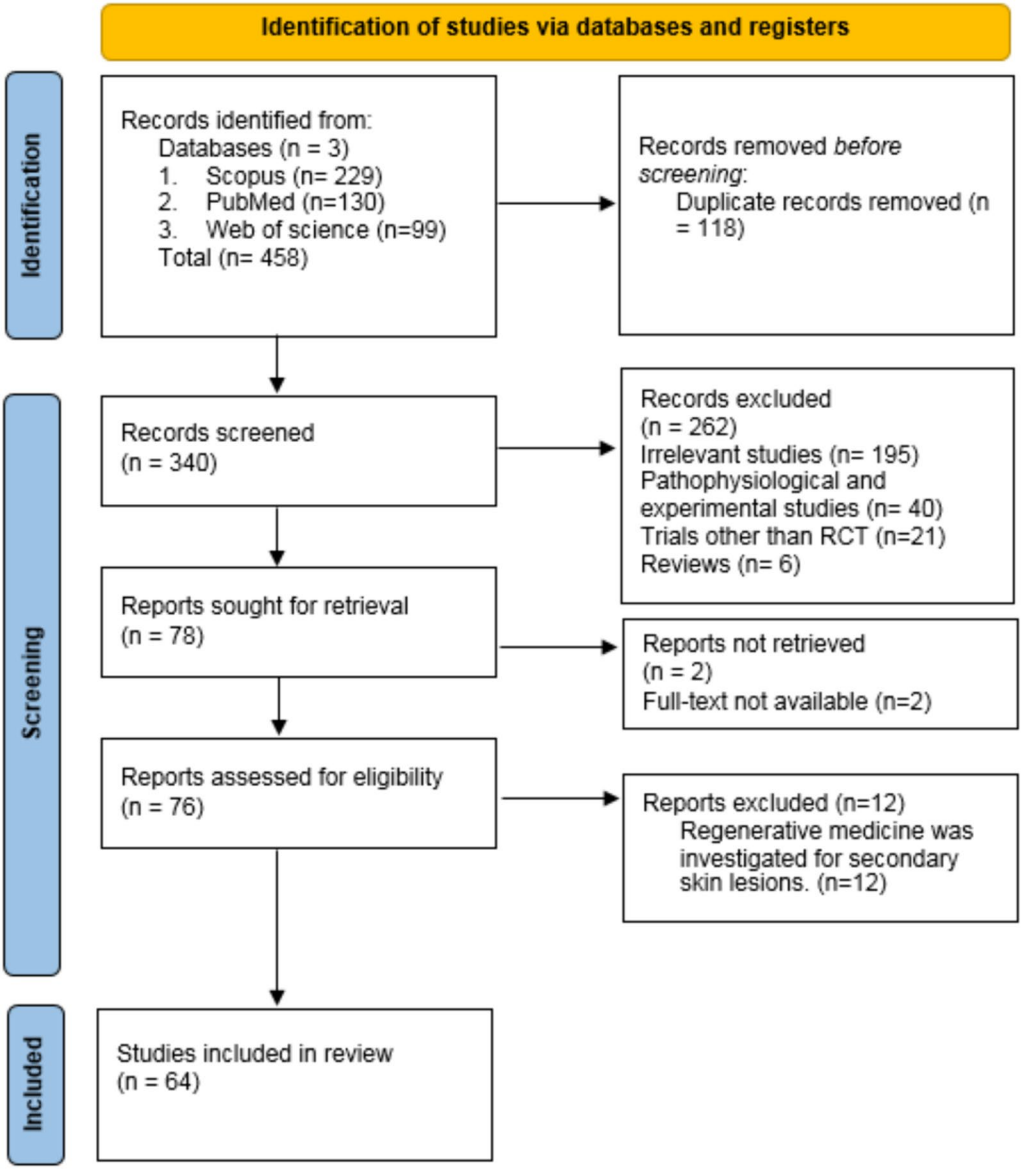
PRISMA chart of studied articles

In our initial search, we obtained 458 articles. Based on the inclusion and exclusion criteria, we selected 65 articles to extract the final data. The screening steps are shown in the PRISMA chart (Fig. 1). The data extracted from the selected studies are presented in Table 2.

**Table 2:** Characteristics of included studies

First author, year	Study design	Study sample size	Age (mean)	Gender ratio (F: M)	Disease type	Type of regenerative medicine	Intervention method	Effectiveness	Adverse events
Gentile et al. [2]	RCT	23 patients	34.74	0:23	AGA	PRP	Three monthly injections of PRP were administered on half of the selected patients’ scalps, while the rest were treated with a placebo	PRP treatment led to a significant increase in hair count, hair density, and terminal hair density compared to the control group, which experienced decreases in these parameters. Differences between the PRP and control groups were statistically significant for all measured parameters	None
Dubin et al. [5]	RCT	30 patients	Unknown	30:0	AGA	PRP	Patients received injections of Eclipse system PRP or saline at three monthly sessions	PRP treatment outperformed saline, with 57% of patients showing significant improvement compared to 7%. After 8 weeks, PRP resulted in a 71.1 hairs/cm2 increase, while the placebo group experienced a -26.7 hairs/cm2 change (*p* < 0.01)	Headache, scalp tightness, swelling, redness, and post-injection bleeding
Alves et al. [19]	RCT	25 patients	39	13:12	AGA	PRP	Patients were split into two groups, Group A and Group B. Group A received PRP treatment on the right side of the head and a placebo on the left side, whereas Group B received PRP on the left side and a placebo on the right side of the head	Six months after the initial PRP treatment, notable changes were observed in average anagen hairs (67.6 ± 13.1), telogen hairs (32.4 ± 13.1), hair density (179.9 ± 62.7), and terminal hair density (165.8 ± 56.8) in comparison to the baseline (*p* < .05)	None
Chuah et al. [20]	RCT	55 patients	38.7 ± 10.5	21:34	AGA	PRP	Participants received PRP injections on one side of the scalp and placebo injections on the other side in four sessions spaced three weeks apart. Follow-up evaluations at 12 and 24 weeks measured changes in hair density and diameter	In all participants, there was a notable rise in hair density at 12 and 24 weeks on the side treated with PRP, with a significant increase observed on the control side only at 24 weeks	Unknown
Siah et al. [21]	RCT	10 patients	35.5	9:1	AGA	PRP	A course of PRP treatment consisting of five sessions over 8 weeks was established based on assessing the changes in hair density and hair thickness in 10 patients affected by Androgenetic Alopecia (AGA)	After 8 weeks following the final PRP injection, the areas that received treatment showed higher average hair density compared to the placebo. The average thickness of hair decreased in both the treated and placebo areas	The side effects are relatively limited
Rodrigues et al. [22]	RCT	26 patients	Unknown	0:26	AGA	PRP	The treatment group was given 4 subcutaneous injections of PRP, while the control group received a saline solution application. Hair growth, density, and the percentage of anagen hairs were assessed 15 days and 3 months after the final injection	The study demonstrated a notable rise in hair count (*P* = 0.0016), hair density (*p* = 0.012), and the percentage of anagen hairs (*p* = 0.007) in the PRP group compared to the control group. This effect was observed regardless of platelet counts or the measurement of growth factors in PRP	None
Tawfik et al. [23]	RCT	30 patients	29.3 ± 6.56	0:30	AGA	PRP	Patients with androgenetic alopecia (AGA) received four autologous PRP injections in one scalp area and placebo injections of normal saline in another area, with one-week intervals between sessions. Follow-up evaluations were performed six months post final treatment	After 6 months, the areas treated with PRP showed a notable improvement in hair density, thickness, and volume compared to the initial measurements with statistical significance (*p* < 0.005)	None
Dicle et al. [24]	RCT	25 patients	30.9	0:25	AGA	PRP	Participants were split into two groups: Group 1 (PRP, n = 10) and Group 2 (saline, n = 15). They each had three sessions of monthly PRP or placebo over 3 months. After 3 months, the groups switched treatments. Hair count was measured at baseline (M0), the 4th month (M4), and the 9th month (M9) for all patients	At the end of the study, both groups showed an increase in total hair count. Group 1 had a 3.4% increase from baseline to M9, while Group 2 had a 6.9% increase. The increase in Group 2 was statistically significant when comparing M4 vs. M9 and M0 vs. M9 (*p* = .014, *p* = .040), but no significant differences were found in Group 1	Local pain
Legiawati et al. [25]	RCT	30 patients	34.5	0:30	AGA	PRP	Patients were divided into two groups: a single-spin group and a double-spin group. The study lasted for six weeks, with visits scheduled every two weeks	The single-spin group showed greater improvements in various aspects such as hair count, hair density, and average hair per unit. On the other hand, the double-spin group demonstrated improvements in increasing the anagen hair rate and decreasing the telogen hair rate, but these findings did not reach statistical significance (*p* > 0.05)	None
Ozcan et al. [26]	RCT	62 patients	33.13	0:62	AGA	PRP	Patients were split into two groups; one group got PRP with microneedling, the other got only PRP. The treatment involved four sessions: three sessions every 2 weeks, followed by a fourth session a month later	The hair-pulling test showed a significant improvement after treatment in both groups. Trichoscan analysis revealed significant changes in both groups as well. A statistically significant difference was observed in the average counts of anagen hair, telogen hair, and hair length between the groups	None
Ramadan et al. [27]	RCT	126 patients	Unknown	80:46	AGA	PRP	All patients received Minoxidil 5%. Group 1 underwent PRP injections every 3–6 months, while Group 2 received microneedling along with PRP sessions. Group 3 served as the control group and did not receive PRP	In terms of clinical improvement scores, the control group had a score of 35.7 ± 14.9, while Group 1 had a score of 64.3 ± 14.3 and Group 2 had a score of 78.3 ± 10.6. Significant enhancements were observed in the PRP-treated groups compared to the control group, with Group 2 exhibiting notably higher improvement than Group 1	Pain and headache
Singh et al. [28]	RCT	86 patients	26.14 ± 3.9	0:86	AGA	PRP	Patients were split into two groups: one group received autologous activated platelet-rich plasma in one half of the affected scalp and autologous non-activated platelet-rich plasma in the other half, with the sequence reversed in the second group. Patient self-assessment and clinical photography were conducted monthly for six months to track progress and outcomes	At each visit, there was a significant contrast (*p* < 0.001) in hair density and thickness between the sections where activators were used and where they were not. The regions treated with activators showed better results compared to those without activators	None
Hausauer et al. [29]	RCT	39 patients	43.75	10:29	AGA	PRP	In a study with twenty participants, two treatment groups were compared regarding subdermal PRP injections. Group 1 had 4 treatments (3 monthly sessions + 1 booster), while Group 2 had 2 treatments (spaced every 3 months)	After 3 months, only Group 1 showed a significant increase in hair count compared to Group 2. By 6 months, both groups had a significant increase in hair count from the baseline. However, Group 1’s treatment protocol was statistically more effective than Group 2, with Group 1 showing a higher mean percentage change in hair count	Pain, discomfort, headache, and itching
Singh et al. [30]	RCT	80 patients	26.1 ± 4.2	Unknown	AGA	PRP	The patients were divided into four groups for the study: Group 1 used topical minoxidil alongside normal saline injections, Group 2 applied minoxidil and received monthly PRP injections for 3 months, Group 3 received normal saline injections for 3 months, and Group 4 applied normal saline and received PRP injections during the study period	The study findings showed that the highest level of patient satisfaction was observed in the group treated with PRP in combination with minoxidil, followed by the PRP-only group, the minoxidil-only group, and the NS group, in decreasing order	None
Bruce et al. [31]	RCT	20 patients	30.77	20:0	AGA	PRP	In Arm A, patients received PRP injections for 3 sessions, followed by an 8-week break and then using minoxidil for 12 weeks. They were monitored for a year. Arm B had the reverse treatment sequence, starting with minoxidil for 12 weeks, followed by an 8-week break, and concluding with PRP treatment	After PRP treatment, there were significant increases in hair count and vellus hair density from baseline to Week 12. On the other hand, minoxidil led to significant increases in hair count, vellus hair density, terminal hair density, and cumulative thickness	Pain, discomfort, and bruising
Gupta et al. [32]	RCT	40 patients	31.5	0:40	AGA	PRP	Group A received a combination of PRP treatment every 15 days and daily oral biotin tablets for three months. In contrast, Group B only received daily oral biotin tablets for the same duration	There was no significant difference in hair regrowth between the two groups after three months. However, by the sixth month and beyond, individuals in Group A showed a statistically significant increase in hair growth compared to Group B, with this difference continuing to progress at the nine and twelve-month marks	Unknown
Sasaki et al. [33]	RCT	8 patients	Unknown	Unknown	AGA	PRP	Participants received platelet-rich plasma (PRP) injections at the beginning of the study and again at the third month. Two different treatment approaches with varying platelet concentrations were administered on the front and top areas of the scalp, while the control areas were injected with normal saline	Both sets of participants demonstrated greater hair density, thicker hair follicles, and an increase in mature hair in the treated regions at the front and top of the scalp compared to the initial measurements. These enhancements were more prevalent when using higher platelet concentrations	None
Balasundaram et al. [34]	RCT	64 patients	26.85	0:64	AGA	PRP	Participants were randomly assigned to receive either Platelet-Rich Plasma (PRP) or Minoxidil treatment. The Minoxidil group received 1 ml of Minoxidil 5% twice a day for six months, while the PRP group underwent three PRP sessions spaced one month apart	At week 12, a notable rise in hair count and density was observed in both groups, with no statistically significant difference between them. The physician assessment revealed a response rate of 56% for Minoxidil and 38% for PRP (*p* = 0.124)	Pain
Padhiar et al. [35]	RCT	62 patients	27.72	0:62	AGA	PRP	Group A patient’s received treatment with 5% topical minoxidil, while Group B patients were administered 5% minoxidil along with platelet-rich plasma therapy every month for six months	In Group A, 25.8% of patients were satisfied, and 6.5% were highly satisfied. In Group B, 35.5% of patients were satisfied, and 29.0% were highly satisfied, showing statistical significance. Additionally, Group B exhibited a significantly higher hair density (42.97 ± 8.96) than Group A (36.94 ± 11.57)	Pain, erythema, and active infections
Mapar et al. [36]	RCT	19 patients	22	0:19	AGA	PRP	Two monthly sessions of PRP were injected at the intervention site, while normal saline was injected at the control site	The mean number of terminal and vellus hairs was 87 and 43 at the beginning of the study, and 85 and 42 at the end of the study, respectively, with no notable change	Unknown
Shapiro et al. [37]	RCT	35 patients	Unknown	Unknown	AGA	PRP	Each patient received either three monthly sessions of PRP injections as the intervention or normal saline injections as part of the control group	In the PRP group, the hair density increased from 151 ± 39.82 hairs/cm2 at baseline to 170.96 ± 37.14 hairs/cm2, while in the placebo group, it increased from 151.04 ± 41.99 to 166.72 ± 37.13 hairs/cm2 (*p* < 0.05). However, there was no significant difference observed in the change of hair density between the two groups (*p* > 0.05)	None
Puig et al. [38]	RCT	26 patients	Unknown	26:0	AGA	PRP	The study investigated the effects of PRP compared to saline when injected into the scalp on hair count, hair mass index (HMI), and patient feedback	No statistically significant differences in average hair count and mass were found between the treatment group and the placebo group	None
Gressenberger et al. [39]	RCT	30 patients	29	0:30	AGA	PRP	Twenty individuals received PRP treatment, while 10 were given saline injections. The PRP group underwent five treatment sessions with platelet-rich plasma administered at 4–6-week intervals, followed by two check-up sessions at 4 weeks (FU1) and 6 months (FU2) after the final treatment	In the PRP group, improvement was reported by 13 patients (68.4%), while 6 individuals (31.6%) did not observe any noticeable change. Within the placebo group, 4 participants (44.4%) experienced improvement, another 4 (44.4%) did not detect any change, and 1 subject (11.1%) reported worsening hair loss	Swelling, Redness, Bleeding in treated areas, Hematoma, and Pain
Wu et al. [40]	RCT	75 patients	Unknown	Unknown	AGA	Combination of PRP and Basic Fibroblast Growth Factor (bFGF)	Participants were allocated randomly into three different groups: Group 1 received a combination of PRP and bFGF (PRPF) injections monthly, Group 2 used Minoxidil 5% twice a day, and Group 3 received a combination of treatments	In the PRPF group, there was a mean hair count increase of 31.28 hairs, in the minoxidil group it was 11.32 hairs, and in the combination therapy group it was 41.96 hairs (*p* < 0.05). The effectiveness of PRPF alone was superior to minoxidil treatment. The combination therapy showed greater efficacy compared to both PRPF alone and minoxidil treatment	pain, redness, and pinpoint bleeding
Lee et al. [6]	RCT	30 patients	Unknown	Unknown	AGA	Conditioned Media	Thirty patients who received non-ablative fractional laser treatment were given either Conditioned Media (CM) or a placebo solution topically	The conditioned media group showed a significant increase in hair density compared to the placebo group. Moreover, the Global Aesthetic Scale (GIS) scores were significantly higher in the CM group than in the placebo group	None
Legiawati et al. [41]	RCT	37 patients	36.67	0:37	AGA	Conditioned Media	The participants were divided into two groups based on the concentration of conditioned media (CM) administered: concentrated and non-concentrated. Each participant’s scalp was divided into two sections, with one section receiving 2 ml of either concentrated or non-concentrated CM, or the other section receiving 2 ml of NaCl	The group receiving non-concentrated CM showed the greatest percentage increases in HC (184.9), HD (185.0), TR (43.4), MT (50.0), and TFU (104.3), along with the largest decrease in VR (-56.0). However, these findings were not statistically significant	Pain, itchiness, and redness
Tak et al. [42]	RCT	38 patients	45.3	9:29	AGA	Adipose-Derived Stem Cell constituents’ extract (ADSC- CE)	Nineteen participants in the intervention group (IG) were treated with ADSC-CE, while nineteen subjects in the control group (CG) received a placebo. Both groups applied a 2 mL solution to the area of hair loss twice daily for 16 weeks	At the 8-week mark, the intervention group (IG) demonstrated a 19.2% increase in hair count compared to the control group (CG). The rise in hair density was significantly notable in IG at 28.1%, in contrast to CG at 7.1%	Unpleasant sensations such as itching, burning, pricking, tingling, and stiffness on the scalp were reported
Tan et al. [43]	RCT	16 patients	30.8 ± 6.6	0:16	AGA	Concentrated Growth Factor (CGF)	Each participant received either CGF or a placebo (normal saline) via intradermal injection in two separate symmetrical scalp areas, during three monthly sessions. Furthermore, all patients received treatment with minoxidil	Combining minoxidil with CGF demonstrated higher effectiveness compared to using minoxidil alone in increasing hair density, the T/V ratio, and the HG ratio. Hair growth was stimulated after 4 weeks in the minoxidil + CGF group, which was earlier than in the group using only Minoxidil	Increased greasy sensation and mild discomfort
Pathania et al. [44]	RCT	20 patients	34.7	0:20	AGA	Preserving hair grafts in PRP	Participants were split into two categories: Hair grafts were stored either in a solution with platelet-rich plasma or chilled Ringer’s lactate, depending on whether they belonged to the experimental or control group	Despite the fact that the proportion of follicular unit grafts (FUGs) in the growth stage was notably higher (*p* value < 0.05) in the PRP group compared to the control group, all patients in both groups achieved over 75% growth after 6 months. The noticeable difference in hair densities between the PRP and non-PRP groups remained statistically significant from 4 weeks to 6 months (*p* < 0.05)	infection
Tegta et al. [3]	RCT	20 patients	21.25	10:10	vitiligo	Transplantation of autologous epidermal melanocyte/keratinocyte cells	In this study, two distinct concentrations of non-cultured epidermal suspension were examined. Group A was administered a suspension sourced from a donor graft roughly one-third the size of the recipient, whereas Group B received a suspension derived from a graft around one-fifth of the recipient’s size	In Group A, with an average of 231.60 ± 27.03 melanocytes/mm2, the repigmentation was more successful compared to Group B, which had an average of 154.90 ± 27.65 melanocytes/mm2. The contrast in repigmentation levels between the two groups at the study’s conclusion was determined to be statistically significant (*p* < 0.05)	bacterial infection at the recipient site
Budania et al. [8]	RCT	41 patients	20.5	26:15	vitiligo	Transplantation of autologous epidermal melanocyte/keratinocyte cells	Participants in group 1 received treatment with non-cultured epidermal suspension (NCES), while those in group 2 underwent treatment with suction blister epidermal graft (SBEG)	Excellent repigmentation scores (indicating 90–100% repigmentation) were observed in 71% of lesions treated with NCES and in 27% of lesions treated with SBEG (*p* = 0.002). Repigmentation exceeding 75% (considered good repigmentation) was noted in 89% of lesions in the NCES group and 85% of lesions in the SBEG group (*p* = 0.61)	None
Gupta et al. [45]	RCT	32 patients	21.03	24:8	vitiligo	Transplantation of autologous epidermal melanocyte/keratinocyte cells	In Group A (comprising 15 participants), the recipient site was prepared using an Er:YAG laser, while in Group B (with 17 participants), preparation was conducted using a motorized dermabrader. Patients from both groups were objectively evaluated for repigmentation after 1, 3, and 6 months	In Group A, a skin surface area of 95.956 cm2 underwent operations, resulting in a repigmentation of 46.758 cm2 (54.7%). In Group B, an area of 157.74 cm2 was operated on, leading to a repigmentation of 78.601 cm2 (48.8%). There was no statistically significant difference in the total repigmentation achieved between the two groups	Hyperpigmentation, scarring, and reactivation of the disease
Jamal-Edine et al. [46]	RCT	19 patients	32.05	12:7	vitiligo	Transplantation of autologous epidermal melanocyte/keratinocyte cells	Each patient had the treated areas divided into two groups at random. One group had a recipient site treated with FCO_2_ laser ablation (fractional group), while the other group had a recipient site treated with full surface CO_2_ laser ablation (full ablation group)	In the study, using FCO_2_ laser resulted in excellent repigmentation in 40% of cases, good repigmentation in 35% of cases, and poor repigmentation in 25% of cases. On the other hand, the full surface CO_2_ laser exhibited excellent repigmentation in 35% of cases and good repigmentation in 30% of cases, while fair repigmentation was seen in 10% to 50% of cases and poor results in 10% to 25% of cases	Depigmentation at the donor site
Hasan et al. [47]	RCT	40 patients	Unknown	Unknown	vitiligo	Transplantation of autologous epidermal melanocyte/keratinocyte cells	The participants were divided into two categories: group A underwent dermabrasion for recipient site preparation, while group B received microneedling. After a period of three months, the patients were evaluated to determine the level of repigmentation	Both dermabrasion and microneedling were effective in achieving repigmentation in the patients. Yet, the participants who received dermabrasion showed a significant and statistically noticeable enhancement compared to the other group	Unknown
van Geel et al. [48]	RCT	28 patients	35.2	14:14	vitiligo	Transplantation of autologous epidermal melanocyte/keratinocyte cells	Following laser ablation, one lesion was treated with a cellular graft enriched with hyaluronic acid, whereas the corresponding lesion was treated with a placebo. Subsequently, all lesions were subjected to UV irradiation twice a week for about two months starting three weeks later	The transplant procedure led to the re-coloring of at least 70% of the treated area in the majority of the vitiligo lesions that were actively treated	None
Toossi et al. [49]	RCT	8 patients	25.75	6:2	vitiligo	Transplantation of autologous epidermal melanocyte/keratinocyte cells	At 8 sites, noncultured MKT was applied, while at 6 sites, dermabrasion was performed without the use of keratinocyte-melanocyte suspension. The degree of repigmentation was evaluated approximately 4 months after the transplantation procedure	Out of the eight vitiligo patches treated, 4 patients (50%) showed an excellent response, 1 patient (12.5%) had a good response, and 3 patients (37.5%) had a poor response to the treatment. No noticeable pigmentation was observed in the control areas	Infection, pain, post-inflammatory hyperpigmentation, and discoloration
Parambath et al. [50]	RCT	21 patients	Unknown	Unknown	vitiligo	Transplantation of autologous epidermal melanocyte/keratinocyte cells and melanocyte–keratinocyte suspension in PRP	Two patches impacted by vitiligo were randomly designated to receive either NCES suspended in PRP or PBS (phosphate-buffered saline)	Following a 6-month duration, the mean repigmentation attained using the area method was 75.6% ± 30% in the PRP group, compared to 65% ± 34% in the group that did not receive PRP. This variance was found to be statistically significant	Unknown
Abdel Halim et al. [51]	RCT	15 patients	Unknown	Unknown	vitiligo	Transplantation of autologous epidermal melanocyte/keratinocyte cells and melanocyte–keratinocyte suspension in PRP	A single lesion was treated with NCECS mixed with PRP, whereas a similar lesion was treated with NCECS in Ringer’s lactate solution. After the treatment, the patients underwent excimer sessions thrice weekly for a duration of three months	Significant enhancements in the size and color of the affected areas were observed in both lesions treated with NCECS combined with PRP and NCECS in Ringer’s lactate. Notably, there was no notable difference in outcomes between the two treatments	Unknown
Razmi et al. [52]	RCT	30 patients	23.4	18:12	vitiligo	Transplantation of autologous epidermal melanocyte/keratinocyte cells and combined cell transplantation with both epidermal and hair follicle origins	After manual dermabrasion, one lesion received epidermal cultured cells while the corresponding anatomically-based lesion of the same patient received a combination of epidermal cultured cells and follicular cultured cells. A blinded observer conducted follow-up assessments at 4, 8, and 16 weeks	The combined therapy showed better results in terms of repigmentation extent (76% vs. 57%, *p* < 0.001), repigmentation speed (48% vs. 31%, *p* = 0.001), color matching (73% vs. 61%, *p* < 0.001), and patient satisfaction	hyperpigmentation at the skin donor
Gunaabalaji et al. [53]	RCT	20 patients	23.9	7:13	vitiligo	Transplantation of autologous epidermal melanocyte/keratinocyte cells and cell transplantation originating from hair follicles	Two vitiligo patches in each patient were randomized to receive one of the two procedures. The patients were followed up for 9 months post-transplantation. The efficacy of hair follicle cell suspension (HFCS) combined with epidermal cell suspension (ECS) in repigmenting leukotrichia and skin in vitiligo was compared	Out of 19 patches treated with HFCS, 10 showed repigmentation of leukotrichia, and out of 19 patches treated with ECS, 8 showed repigmentation (without a significant difference between the groups).@@In the ECS group, 70% of patches achieved > 75% repigmentation, while in the HFCS group, 50% showed > 75% repigmentation of the vitiligo patch	Pain and discharge
Singh et al. [54]	RCT	30 patients	22	20:10	vitiligo	Transplantation of autologous epidermal melanocyte/keratinocyte cells and cell transplantation originating from hair follicles	Thirty patients with 47 stable vitiligo lesions were divided into two groups. Group 1 received treatment with NCES, while Group 2 received treatment with cell transplantation originating from hair follicles. They were assessed for the level of repigmentation and color match on day 8, as well as at weeks 4, 8, 12, and 16 after the surgery	In group 1, 20 out of 24 lesions (83%) exhibited excellent repigmentation, whereas in group 2, 15 out of 23 lesions (65%) showed similar results. However, this difference did not reach statistical significance	None
Mrigpuri et al. [55]	RCT	30 patients	24.23 ± 5.81	16:14	vitiligo	Transplantation of autologous epidermal melanocyte/keratinocyte cells	The participants in the research were randomly divided into two groups: one group received NCES made using the 4C technique, while the other group received NCES prepared in the lab (lab-NCES). An impartial observer assessed each patient at 4, 8, and 16 weeks post-surgery	The outcomes for repigmentation were comparable between the 4C method and lab-NCES. Both methods yielded similar results, with 34% achieving excellent repigmentation (≥ 90%) for the 4C method and 37% for lab-NCES. The difference between the two groups was not statistically significant (*p* = 1.000)	surgical site infection
Deng et al. [56]	RCT	60 patients	40.35	34:26	vitiligo	PRP	Participants were assigned randomly to three distinct treatment categories. Group I was provided with PRP injections directly into the skin, Group II underwent treatment with the 308-nm excimer laser exclusively, and Group III received a combination of the 308-nm excimer laser along with PRP injections. The various treatments lasted for a period of 3 months	Group III showed the highest score, followed by Group II, and then Group I. When it came to repigmentation responses, significant differences were observed among the groups (*p* < 0.001), with Group III showing the most favorable outcome	None
Khattab et al. [57]	RCT	52 patients	25.16	44:8	vitiligo	PRP	Group I, comprising 26 patients, underwent a treatment regimen involving intradermal PRP injection in conjunction with the excimer laser. In contrast, Group II, also comprising 26 patients, received treatment solely involving the excimer laser	Group I showed a significantly higher rate of response to treatment than group II, with a statistically meaningful variance. Moreover, there was a notable variation in Visual Analog Scale (VAS) scores between the two groups (*p* < .000)	Pain and erythema
Afify et al. [58]	RCT	20 patients	46.5 ± 14.5	11:9	vitiligo	PRP	Participants in each treatment group were allocated randomly to receive one of the following interventions: fractional CO_2_ laser, PRP, a combination of fractional CO_2_ with PRP, a combination of fractional CO_2_ with NB-UVB, a combination of fractional CO_2_ with PRP and NB-UVB, or no treatment as part of the control group	A substantial and statistically significant enhancement was observed in the group that received CO_2_ laser with PRP (*p* = 0.001) as well as in the group that received CO_2_ laser with PRP and NB-UVB (*p* = 0.001)	Erythema
Abdelghani et al. [59]	RCT	80 patients	33.83	50:30	vitiligo	PRP	Patients were divided into four treatment groups: fractional CO_2_ laser, PRP, a combination of fractional CO_2_ laser and PRP, and a combination of fractional CO_2_ laser and NB-UVB. The duration of the treatment was 2 months, and patients underwent clinical assessment 3 months after completing the final treatment session	The group that underwent laser treatment and PRP had the most favorable outcomes in terms of repigmentation and patient satisfaction. The use of fractional CO_2_ laser in combination with PRP injection shows promise as a treatment for vitiligo, with the combination of fractional CO_2_ laser and NB-UVB phototherapy following closely behind	None
Sahni et al. [60]	RCT	25 patients	26.48	11:14	vitiligo	Isolated melanocyte transplantation	The research examined the repigmentation results of two groups: Group A, which consisted of 13 patients with 18 lesions who received melanocytes suspended in normal saline, and Group B, which included 12 patients with 18 lesions who received melanocytes suspended in their own serum	Repigmentation was rated as excellent (> 90%) and very good to excellent (> 75%) in 44.4% and 66.7% of lesions, respectively, in Group A, and in 88.8% and 94.4% of lesions, respectively, in Group B	Halo phenomenon, infection at the recipient site, hyperpigmentation, and scarring
Zhang et al. [61]	RCT	473 patients	22.5	244:229	vitiligo	Isolated melanocyte transplantation	Patients receiving cultured autologous melanocyte transplantation were split into four study groups randomly. Group 1 had 20 sessions of NB-UVB treatment before transplantation, Group 2 had 30 sessions after transplantation, Group 3 had 20 sessions before and 30 after, and Group 4 only had transplantation without extra treatment	Group 3 showed the most favorable response rate. Statistical analysis indicated a noteworthy difference among the four groups (χ^2^ = 35.56, *p* < 0.001)	None
Vashisht et al. [62]	RCT	22 patients	Unknown	Unknown	vitiligo	Cell transplantation originating from hair follicles	Three comparable patches underwent random allocation to three separate treatments for hair follicle cell suspension: (A) trypsin combined with collagenase, (B) only trypsin, and (C) dermabrasion using just vehicle. The evaluation of repigmentation was conducted throughout a 6-month follow-up period	The repigmentation percentages observed in the groups were: group A (33.22%) > group B (24.31%) > group C (16.59%); and the corresponding *p*-value was 0.13. It was found that the addition of collagenase led to notably higher repigmentation compared to using dermabrasion alone, with a statistical significance level of *p* < 0.05	Unknown
Khan et al. [63]	RCT	60 patients	20.04	35:25	Alopecia areata	PRP	Group A received injections of triamcinolone intradermally over four monthly sessions, while Group B received PRP injections using the same schedule	SALT score: The effectiveness of the steroid injection was notably superior to that of platelet-rich plasma (*p* < 0.05). Group A demonstrated an effective treatment response in 90% of cases (*p* < 0.05), whereas Group B showed a 73% efficacy rate (*p* = 0.05)	Unknown
Hegde et al. [64]	RCT	50 patients	Unknown	Unknown	Alopecia areata	PRP	The participants were evenly split into group P (PRP) and group S (steroid). Each participant received a total of 3 treatment sessions, with each session scheduled every 4 weeks	Both treatment groups demonstrated significant improvement in the SALT score (*p* < 0.001). The highest absolute hair regrowth was observed in the steroid group, followed by PRP, and then the placebo group (*p* = 0.016)	Pain
Trink et al. [65]	RCT	45 patients	28	25:20	Alopecia areata	PRP	The patients were split into three groups: PRP, triamcinolone acetonide (TrA, 2.5 mg/ml), or a placebo. Each patient received three treatments, spaced one month apart	The use of both TrA and PRP resulted in a notable increase in hair regrowth compared to the placebo. Additionally, both treatments showed enhanced hair regrowth compared to the untreated area of the scalp. Furthermore, patients receiving PRP alone experienced significantly greater hair regrowth compared to those treated with TrA	None
Ragab et al. [66]	RCT	60 patients	31.8	12:48	Alopecia areata	PRP	Patients were divided into three groups of equal size through random selection. Each group underwent monthly sessions of either PRP injections, fractional CO_2_ laser (FCO_2_) followed by topical PRP, or microneedling followed by topical PRP for three consecutive months	In groups A, B, and C, 80%, 80%, and 70% of participants experienced improvement, respectively. There was no significant difference in the degree of improvement between the groups based on physicians’ assessment (*p* = 0.268) or patient satisfaction (*p* = 0.147)	Pain, erythema, edema, itching, ecchymosis, and headache
El Taieb et al. [67]	RCT	90 patients	21.09	51:39	Alopecia areata	PRP	Patients were randomized into three groups: the first group received topical minoxidil 5% twice daily, the second group received PRP injections in three monthly sessions, and the third group used topical panthenol cream twice daily as a placebo	Both minoxidil 5% and PRP showed noticeably greater hair growth results compared to the placebo (*p* < 0.05). PRP demonstrated an earlier response with hair regrowth, reduction in short vellus hair, and dystrophic hair compared to minoxidil and the control group (p < 0.05)	Unknown
Gupta et al. [10]	RCT	27 patients	Unknown	Unknown	Alopecia areata	PRP	Alopecia patches on both sides of the scalp were randomly selected to receive either 3 intradermal injections of platelet-rich plasma or normal saline at monthly intervals, and were assessed 3 months after the final treatment session	The mean Severity of Alopecia Tool score did not change significantly in either group. However, the mean percentage reduction in the score was higher in the PRP group compared to the placebo group (9.05% ± 36.48% vs 4.99% ± 33.88%; *p* = 0.049)	Unknown
Sirithanabadeekul et al. [68]	RCT	10 patients	46.2	10:0	Melasma	PRP	Four bi-weekly sessions of PRP were injected intradermally on one side of the face (PRP side) and normal saline on the other side (control side)	The average levels of melanin and wrinkles showed significant changes (*p* = 0.038 and *p* = 0.040) in the PRP group compared to the control group. The mean mMASI score indicated a significant improvement only in the PRP group (*p* = 0.042). However, there was no significant change in the melanin index in either group	Bruising
Bikash et al. [12]	RCT	30 patients	Unknown	Unknown	Melasma	PRP	Thirty individuals were selected at random to undergo PRP microinjections on one side and normal saline on the other side across three monthly sessions. All patients were instructed to apply 4% HQ cream on both treated areas	53.3% of participants in the PRP + HQ group and 76.7% in the HQ group saw a 25–50% enhancement in their MASI scores. Additionally, 40% in the PRP + HQ group witnessed a 51–75% improvement, which was much higher than the mere 3.3% in the HQ group. This variance was deemed statistically significant	Unknown
Adelipour et al. [69]	RCT	50 patients	27.93	50:0	Melasma	Topical use of umbilical cord as a source of mesenchymal stem cells	Fifty expectant mothers were randomly divided into two groups: one served as the control group without any intervention, while the other received treatment involving the umbilical cord	MASI: In the intervention group, the values changed from 5 ± 0.66 at baseline to 0.77 ± 0.22 one month after treatment with the umbilical cord. For the control group, the measurements shifted from 4.26 ± 0.69 at baseline to 4.1 ± 0.68. A significant difference was observed between the individuals in the treatment group before and after receiving the umbilical cord therapy	None
Goldstein et al. [14]	RCT	29 patients	52.6	29:0	LSA	PRP	Out of the total, ten patients were given a placebo (saline injections), whereas PRP was administered to 19 patients across two sessions spaced six weeks apart. The effectiveness was evaluated through histology and the Clinical Scoring System for Vulvar Lichen Sclerosus (CSS)	In the PRP group, 5 patients displayed improvement in histopathologic inflammation, while 10 showed no change, and 4 exhibited increased inflammation. For women who received the placebo, 5 experienced improvement, 4 saw no change, and 1 displayed increased inflammation (*p*-value = 0.542)	Bruising
Tedesco et al. [70]	RCT	40 patients	43	16:24	LSA	SVF and PRP	Patients were divided into two groups: one receiving SVF and the other receiving SVF plus PRP. They were clinically evaluated and had their Dermatology Life Quality Index (DLQI) assessed before and 6 months after treatment	SVF and SVF combined with PRP are proven to be safe and efficient treatments for LSA when administered before patients advance to a severe advanced stage. The individual rates of cellular proliferation assessed in keratinocytes, melanocytes, fibroblasts, and AD-MSCs did not exhibit any notable relationship with treatment effectiveness as assessed by clinical scores or DLQI	None
Moftah et al. [13]	RCT	30 patients	23.73 ± 5.77	26:4	Inflammatory acne vulgaris	PRP	The patients were divided into two groups. One group underwent four sessions of PRP injections within the skin lesions on one side of the face, while the other group received treatments using a long-pulsed Nd:YAG laser (1064 nm) on the opposite side, with a 2-week interval between sessions	The findings indicated that both approaches were highly effective in decreasing active acne lesions. Nonetheless, there was no notable contrast in the recovery of lesions between the two groups. The group that underwent PRP treatment experienced a substantial 58.77% decrease in inflammatory acne lesions	None
El-Dawla et al. [11]	RCT	30 patients	29.17	30:0	Chronic telogen effluvium	PRP	Group 1 was given PRP generated with specialized kits, Group 2 was administered PRP prepared using standard laboratory tubes, and Group 3 was provided with a placebo of normal saline solution. Patients underwent four rounds of intradermal injections at four-week intervals	The results of the hair pull test and visual analog scale indicated a notable difference between Group 1 and Group 3, as well as between Group 2 and Group 3. Patient satisfaction levels also demonstrated a significant variance between Group 1 and Group 3, as well as between Group 2 and Group 3, with no discernible distinction between Group 1 and Group 2	Headache
Hijazi et al. [15]	RCT	20 patients	46.45	18:2	Erosive Oral lichen planus	PRP	Patients were divided into two groups: PRP or triamcinolone (TA). They received weekly intralesional injections for 4 weeks, followed by a three-month follow-up with regular visits every two weeks. During the follow-up sessions, the numerical pain scale, clinical picture, and remission time were evaluated	Eighty percent of patients in the PRP group showed complete remission, compared to 70% in the TA group. Pain scores at the end of treatment in both groups were statistically significant (*p* = 0.010). Similar results were recorded in clinical scores (*p* = 0.042). By Week 17, both groups showed a significant reduction in scores, with no statistically significant difference between the two groups	None
El-Darouti et al. (17)	RCT	14 patients	5.75	8:6	Dystrophic epidermolysis bullosa	Intravenous injection of mesenchymal stem cells	The patients were divided into two groups. Both groups received intravenous MSC (derived from bone marrow aspiration from one healthy parent). Group 1 also received cyclosporine suspension at a dose of 5 mg/kg per day, while Group 2 received a placebo	In both groups, there was a significant decrease in the number of new blisters (*p* = 0.003 and 0.004, respectively). The healing rate of new blisters significantly accelerated in both groups (*p* < 0.001), with no notable difference between the two groups	None

RCT, Randomized Controlled Trial; AGA, Androgenetic Alopecia; PRP, Platelet-Rich Plasma; SVF, Stromal Vascular Fraction; bFGF, Basic Fibroblast Growth Factor; ADSC-CE, Adipose-Derived Stem Cell Constituents’ Extract; CGF, Concentrated Growth Factor; NCES, Non-Cultured Epidermal Suspension; SBEG, Suction Blister Epidermal Graft; FCO_2_, Fractional CO_2_ Laser; NB-UVB, Narrowband Ultraviolet B; HQ, Hydroquinone; MASI, Melasma Area and Severity Index; DLQI, Dermatology Life Quality Index; SALT, Severity of Alopecia Tool; VAS, Visual Analog Scale; MKT, Melanocyte–Keratinocyte Transplantation; HFCS, Hair Follicle Cell Suspension; ECS, Epidermal Cell Suspension; TA, Triamcinolone Acetonide; MSC, Mesenchymal Stem Cells

In a total of 64 studies, 2888 patients with dermatologic diseases were examined. Among them, 1086 were women (44.8%) and 1339 were men (55.2%). However, in 12 studies (18.2% of the studies involving 463 patients), the separation of gender was not conducted.

Out of the 64 studies, the average age of the participants was reported in 49 studies, with the average age of the patients being 27.46 years. All 64 studies were randomized controlled trials (RCTs). Among the 64 studies, 29 studies (45.3%) focused on androgenetic alopecia (AGA), 20 studies (31.2%) on vitiligo, 6 studies (9.3%) on alopecia areata, 3 studies (4.7%) on melasma, 2 studies (3.1%) on lichen sclerosis et atrophicus (LSA), and 1 study (each) on inflammatory acne vulgaris, chronic telogen effluvium, erosive oral lichen planus, and dystrophic epidermolysis bullosa.

Among the 64 studies conducted, there were a total of 149 intervention groups, with 102 groups treated using regenerative medicine methods. The following information provides the number and percentage of utilization for each regenerative medicine method:

- Platelet-rich plasma (PRP) injection in 57 groups (55.9%).
- Transplantation of autologous epidermal melanocyte/keratinocyte cells in 19 groups (18.6%).
- Isolated melanocyte transplantation in 6 groups (5.9%).
- Cell transplantation with hair follicle origin in 5 groups (5%).
- Melanocyte–keratinocyte suspension in PRP in 2 groups (1.9%).
- Conditioned media injection in 2 groups (1.9%).
- Combination of PRP and Basic fibroblast growth factor (bFGF) in 2 groups (1.9%).
- Intra venous injection of mesenchymal stem cells in 2 groups (1.9%).
- Concentrated growth factor (CGF) in 1 group (1%).
- Stromal vascular fraction (SVF) in 1 group (1%).
- Combination of PRP and SVF in 1 group (1%).
- Preserving hair grafts in PRP in 1 group (1%).
- Topical use of umbilical cord as a source of mesenchymal stem cells in 1 group (1%).
- Adipose-derived stem cell constituents extract in 1 group (1%).
- Combined cell transplantation with epidermal and hair follicle origin in 1 group (1%).

Adverse events were reported in 29 studies (45.3%), with the most common side effect being pain during the procedure. All side effects mentioned in the studies were mild, and no serious or life-threatening side effects were reported.

### Androgenetic alopecia (AGA)

Of the 2888 patients included in the study, 1190 patients (41.2%) were diagnosed with AGA and received interventions. The most commonly used regenerative medicine method in this group was PRP, which was utilized in 36 intervention groups (from 23 studies). Among 23 studies, significant effectiveness of PRP compared to the control group has been proven in 19 studies, with no significant difference reported in the remaining 4 studies.

Additionally, the combination of PRP and Basic Fibroblast Growth Factor (bFGF), as well as Conditioned Media, were used in two intervention groups each (2 studies on conditioned media and one study on the combination of PRP and bFGF). Concentrated Growth Factor (CGF), Adipose-Derived Stem Cell constituents’ extract, and preserving hair grafts in PRP were each studied in one group and one study.

#### PRP

In several studies, the effectiveness of PRP in treating AGA has been reported. Monthly intradermal injections of PRP for three months, on average, resulted in an increase of 33.6 hairs in the treated area, while the placebo area experienced a decrease of 3.2 hairs. The PRP-treated group showed a significant difference in hair density compared to the placebo group, with an increase of 45.9 hairs per square centimeter versus a decrease of 3.8 hairs per square centimeter. Moreover, the density of terminal hairs increased by 40.1 hairs per square centimeter in the PRP-treated group, which was significantly different from the control group. However, there was no significant difference in the density of vellus hairs compared to the placebo group [2].

In another similar study, PRP injections at one-month intervals for three months improved average hair density by 71.1 hairs per square centimeter in the PRP-injected area, while the placebo group experienced a decrease of 26.7 hairs per square centimeter. By the end of the eighth week, the PRP-treated group showed a density improvement of 105.9 hairs per square centimeter, contrasting with a reduction of 52.4 hairs per square centimeter in the placebo group. This difference remained significant between the two groups [5].

In another study, the effectiveness of three monthly PRP injections was compared to a placebo. The results confirmed a significant improvement in anagen hair (67.6 ± 13.1), telogen hair (32.4 ± 13.1), and hair density (179.9 ± 62.7). Additionally, there was an improvement in the density of terminal hair, which was found to be 165.8 ± 56.8 [19]. Treatment with PRP for four sessions at three-week intervals led to a significant improvement in hair density during weeks 12 and 24 following the treatment. It is noteworthy to mention that in the placebo group, a significant difference in hair density compared to the intervention was only observed during week 24 [20].

In a study where patients underwent five sessions of PRP treatment within eight weeks and were evaluated eight weeks after the last treatment session, the intervention group showed an average hair density increase of 12.76%, while the placebo group reported a hair density increase of only 0.99%. This significant difference between the two groups indicates the effectiveness of the intervention. However, it is worth noting that hair caliber decreased in both groups [21]. Additionally, in other studies, significant effectiveness of PRP has been demonstrated compared to control groups and standard treatments [22–35] (Table 2).

However, it is important to note that in several studies, no significant response has been reported after PRP treatment. For example, PRP injection twice with a one-month interval did not cause significant differences compared to the placebo group [36]. Similarly, injections with intervals of one month for three sessions led to improvement in hair density, but did not show a significant difference compared to the placebo group [37]. These results have been repeated in other studies where PRP injection, while controlling hair loss and increasing hair thickness and regrowth in some patients, did not cause a significant difference compared to the placebo group [38].

Another similar study, in which patients underwent five sessions of PRP with a 4–6 week interval and were evaluated one month and six months later, showed no significant difference in the number of hairs, hair diameter, and patient satisfaction between the intervention and control groups [39].

#### Combination of PRP and basic fibroblast growth factor (bFGF)

In the single study examining the use of both PRP and basic fibroblast growth factor (bFGF) in treating AGA, the findings demonstrated a notable enhancement in the group that received this combination alongside 5% topical minoxidil, in contrast to the group that only received the PRP and bFGF combination. Moreover, there was a significant improvement seen in the group treated with the PRP and bFGF combination compared to the group solely using 5% topical minoxidil. The average increase in hair count was approximately 31.28 hairs in the PRP group, 11.32 hairs in the minoxidil group, and 41.96 hairs in the combined therapy group (*p* < 0.05) [40].

#### Conditioned media

In both studies investigating the use of adipose-derived mesenchymal stem cell-conditioned medium in AGA treatment, a significant improvement has been demonstrated compared to the control group in terms of the percentage increase in Hair Count (184.9%), Hair Density (185.0%), Terminal rate (43.4%), Mean thickness (50.0%), and Total follicular units (104.3%). The studies also showed the highest decline in Vellus rate (− 56.0%) [6, 41].

#### Adipose-derived stem cell constituents’ extract

In the only study investigating the effectiveness of adipose-derived stem cell constituents extract (ADSC-CE) in the treatment of AGA, a significant improvement in hair count, hair density in the 8th week of treatment, and hair diameter in the 16th week was reported compared to the control group. A 19.2% increase in hair count and a 21% increase in hair density, contrasting with the control group, were observed [42].

#### Concentrated growth factor (CGF)

In the only study investigating the effectiveness of concentrated growth factor (CGF) in the treatment of AGA, the results showed a significant improvement in hair density, the ratio of terminal hairs to vellus, and the amount of hair growth compared to the control group. Furthermore, the satisfaction level of patients in the group receiving the combination of minoxidil and CGF was reported as 75% [43].

#### Preserving hair grafts in PRP

In the only study investigating the preservation of hair grafts using PRP to enhance hair transplantation results, the findings showed a substantial increase in hair density within the PRP group compared to the control group over a 6-month period. All patients demonstrated over 75% hair growth [44].

### Vitiligo

Out of the 2888 patients included in the study, 1087 patients (37.6%) were diagnosed with vitiligo and underwent interventions. The most commonly used regenerative medicine method in this group was the transplantation of autologous epidermal melanocyte/keratinocyte cells, which was employed in 19 intervention groups from 13 studies. In all 13 studies investigating the transplantation of autologous epidermal melanocyte/keratinocyte cells, a significant improvement in clinical response was reported.

Subsequently, PRP was utilized in 8 intervention groups across 4 studies. Isolated melanocyte transplantation was employed in 6 intervention groups across two studies. Cell transplantation originating from hair follicles was utilized in 5 intervention groups across 3 studies (including 2 joint studies with the transplantation of autologous epidermal melanocyte/keratinocyte cells). Melanocyte–keratinocyte suspension in PRP was employed in 2 intervention groups across 2 studies (both studies were conducted in conjunction with the transplantation of autologous epidermal melanocyte/keratinocyte cells). Lastly, combined cell transplantation with both epidermal and hair follicle origins was utilized in a single intervention group in a study (a joint study with the transplantation of autologous epidermal melanocyte/keratinocyte cells).

#### Transplantation of autologous epidermal melanocyte/keratinocyte cells

In a study comparing cell transplantation from grafts with a size of 1/3 of the recipient area, with a cell count of 231.60 ± 27.03 melanocytes per square millimeter, to grafts with a size of 1/5 of the recipient area, with a cell count of 154.90 ± 27.65, the amount of repigmentation and color match in the first group were significantly better than in the second group. However, the time of the onset of repigmentation was not significantly different between the two groups [3].

In a study comparing two methods of suspending autologous epidermal melanocyte/keratinocyte cells and grafting through suction blister, both groups demonstrated significant improvement. However, the transplantation through suspension showed a more remarkable improvement compared to the suction blister group (*p* = 0.002). An excellent response was observed in 71% of lesions in the epidermal melanocyte/keratinocyte group, while only 27% of the lesions in the suction blister group exhibited such a response [8].

Regarding the preparation of the recipient site of autologous epidermal melanocyte/keratinocyte cells, site preparation with Erbium YAG laser has resulted in 54.7% repigmentation, while preparation with motorized dermabrasion has led to 48.8% repigmentation; both methods show significant improvement. However, no significant difference was observed between the two groups [45].

Also, in the comparison of the preparation of the receptor site between fractional CO_2_ and full surface CO_2_ methods, excellent repigmentation was observed in 40% and 35% of patients, respectively. Despite the significant improvement in both groups, no significant difference has been noted between the two groups [46]. Furthermore, in the comparison of the two methods of dermabrasion and microneedling at the transplant site, both methods have shown effectiveness. However, dermabrasion has demonstrated significant improvement compared to microneedling [47].

The results of other investigated studies have also confirmed the significant effectiveness of transplantation of autologous epidermal melanocyte/keratinocyte cells compared to the control group and those receiving standard treatment [48–55] (Table 2).

#### PRP

In all four studies investigating the effectiveness of PRP in treating vitiligo, significant improvement has been observed. In one study, the combination of excimer laser and PRP resulted in a significant improvement compared to each method alone. Additionally, in this study, excimer laser was significantly more effective than PRP in healing lesions [56]. In another similar study, the combination of excimer laser and PRP was significantly more effective than excimer laser alone [57].

In a study comparing PRP, fractional CO_2_ laser, the combination of these two methods, the combination of fractional CO_2_ laser and UVB, and the combination of PRP, fractional CO_2_ laser, and UVB in the treatment of vitiligo, the results showed a significant difference in the rate of recovery in all aforementioned groups compared to the control group. However, there was no significant difference between the intervention groups [58].

In a study comparing fractional CO_2_ laser and PRP, the combination of the two, and the combination of fractional CO_2_ laser and UVB in the treatment of vitiligo, the results demonstrated the superiority of the combination of fractional CO_2_ laser and PRP, followed by the combination of fractional CO_2_ laser and UVB, and finally fractional CO_2_ laser and PRP individually. These differences were found to be significant [59].

#### Isolated melanocyte transplantation

In both studies investigating isolated melanocyte transplantation for the treatment of vitiligo, significant improvements were observed in the lesions.

In one study, melanocyte suspension in the individual’s own serum was compared with melanocyte suspension in normal saline for the treatment of vitiligo. The results showed a significant improvement in both methods, with the first group showing a significantly greater improvement than the second group [60].

In another study evaluating melanocyte transplantation for the treatment of vitiligo, patients were divided into four groups. The first group received melanocyte transplantation and underwent 20 sessions of UVB before transplantation. The second group underwent transplantation and received 30 sessions of UVB after transplantation. The third group received 20 sessions of UVB before transplantation and 30 sessions of UVB after transplantation. The fourth group only underwent transplantation.

The results demonstrated a significant improvement in groups 1 to 3 compared to group 4. Additionally, there was a significant improvement in group 3 compared to groups 1 and 2. However, there was no significant difference in the amount of improvement between groups 1 and 2 [61]**.**

#### Cell transplantation originating from hair follicles

In all three studies investigating the effectiveness of cell transplantation with hair follicle origin for the treatment of vitiligo, significant improvement has been observed in the lesions.

In a study examining three methods for cell transplantation with hair follicle origin in vitiligo treatment—using trypsin and collagenase, using trypsin alone, and using dermabrasion alone—the results showed a significant improvement in the first method compared to the third method. However, no significant difference was observed between the first and second methods, as well as between the second and third methods [62].

In two studies comparing cell transplantation with hair follicle origin and autologous epidermal melanocyte/keratinocyte cell transplantation for improving vitiligo lesions, both methods resulted in significant improvement. However, there was no significant difference in the degree of improvement between the two methods [53, 54].

#### Melanocyte–keratinocyte suspension in PRP

In two studies comparing the transplantation methods of Melanocyte–keratinocyte suspension in PRP and the usual melanocyte/keratinocyte suspension, both methods demonstrated significant improvement. In one study, there was a significant improvement reported in the Melanocyte–keratinocyte suspension in PRP method compared to the usual melanocyte/keratinocyte suspension [50], while the second study reported no significant difference between the two groups [51].

#### Combined cell transplantation with both epidermal and hair follicle origins

In a study comparing the combination of two methods—transplantation of autologous epidermal melanocyte/keratinocyte cells and cell transplantation with hair follicle origin—with transplantation of autologous epidermal melanocyte/keratinocyte cells alone, the results showed a significant increase in repigmentation amount, speed, and color match in the first group compared to the second group [52].

### Alopecia areata

Out of the 2888 patients included in the study, 332 patients (15.9%) were diagnosed with alopecia areata and received interventions. The most commonly utilized regenerative medicine method in this group was PRP, which was used in all seven intervention groups across six studies.

Out of the six studies examining PRP in the treatment of alopecia areata, five studies reported a significant improvement with it, while one study indicated no significant difference in the amount of improvement compared to the control group.

#### PRP

In three studies comparing PRP and triamcinolone acetonide injection for healing lesions, both methods showed significant improvement. However, in two studies, triamcinolone demonstrated greater improvement compared to PRP [63, 64], while in one study, PRP showed significant improvement compared to triamcinolone [65].

In a study comparing three methods of PRP, including PRP injection, the combination of fractional CO_2_ laser and PRP, and the combination of PRP and microneedling for lesion healing, all three methods demonstrated significant improvement, without any notable differences among them [66].

Furthermore, in a study comparing PRP and topical minoxidil 5% for the treatment of alopecia areata, both groups exhibited significant improvement, with PRP showing a significantly faster response in the beginning of hair regrowth [67].

Also, in a study that compared 3 sessions of PRP injections with a one-month interval to normal saline injections for the treatment of alopecia areata, the results showed a decrease in the severity of the disease by 9.05% in the PRP group and 4.99% in the normal saline group. There was no significant difference [10].

### Melasma

Out of the 2888 patients included in the study, 90 patients (3.1%) were diagnosed with melasma and received interventions. The most commonly utilized regenerative medicine method in this group was PRP, which was used in two intervention groups across two studies. Additionally, the topical use of umbilical cord as a source of mesenchymal stem cells has been evaluated in an intervention group through a study.

#### PRP

In both studies investigating the effectiveness of PRP in improving melasma, the results showed a significant difference in the improvement rate compared to the control group. In the first study, the changes in the mean melanin level and mean mMASI score in the group receiving PRP for 4 sessions with a 2-week interval were significantly higher than those in the group receiving normal saline [68]. In the second study, the results indicated a good response in 53.3% of patients receiving the combination of hydroquinone and PRP compared to 27% of patients receiving hydroquinone alone, and this difference was statistically significant [12].

#### Topical use of umbilical cord as a source of mesenchymal stem cells

In the only study that used umbilical cord as a skin mask rich in mesenchymal stem cells, the results demonstrated a significant improvement compared to the control group. The MASI score showed a decrease of 4.23 points in the intervention group and a decrease of 0.16 points in the control group, and this difference was statistically significant [69].

#### Lichen sclerosis et atrophicus (LSA)

Of the 2888 patients participating in the study, 69 patients (2.4%) were diagnosed with LSA and received interventions. Among the studies, PRP was investigated as an intervention in one study, and PRP and SVF were investigated as interventions in another study.

#### PRP

The only study examining PRP in the treatment of LSA found no significant improvement in the intervention group compared to the control group. The changes in the clinical scoring system for vulvar lichen sclerosus (CSS) in the group receiving PRP were -7.74, while in the placebo group they were -9.44. These changes were not significant between the two groups [14].

#### SVF

In a study comparing the combination of SVF and PRP to SVF alone, both methods were found to be significantly effective in healing lesions, but no significant difference was observed between the two groups [70].

### Inflammatory acne vulgaris

Out of the 2888 patients included in the study, 30 patients (1%) were diagnosed with inflammatory acne and received interventions. The only modality investigated in this group was PRP injection.

#### PRP

The only study conducted on regenerative medicine in the treatment of inflammatory acne focused on comparing PRP and Long Pulsed Nd YAG Laser. The results showed significant effectiveness of both methods in reducing active acne lesions. However, no significant difference was observed in the healing of lesions between the two groups. In the group treated with PRP, a 58.77% reduction in the number of inflammatory acne lesions was seen [13].

### Chronic telogen effluvium

Out of the 2888 patients included in the study, 30 patients (1%) were diagnosed with chronic telogen effluvium and received interventions. The only modality investigated in this group was PRP injection.

#### PRP

The only study conducted on regenerative medicine in the treatment of chronic telogen effluvium focused on comparing two methods of PRP (platelet-rich plasma) preparation. The first method involved preparing PRP using a centrifuge at 3500 rpm for 10 min, while the second method utilized a centrifuge at 1000 rpm for 10 min.

The results demonstrated the significant effectiveness of both methods in controlling hair loss, increasing hair density and thickness, as well as improving patient satisfaction. However, no significant difference was observed between the two groups [11].

### Erosive oral lichen planus

Out of the 2888 patients included in the study, 20 patients (0.7%) were diagnosed with erosive oral lichen planus and received interventions. The only modality investigated in this group was PRP injection.

#### PRP

The only study conducted to investigate regenerative medicine in the treatment of erosive oral lichen planus focused on comparing PRP and triamcinolone injection. The results indicated the significant effectiveness of both methods in healing the lesions, and no significant difference was observed between the two groups [15].

### Dystrophic epidermolysis bullosa

Out of the 2888 patients included in the study, 14 patients (0.5%) were diagnosed with dystrophic epidermolysis bullosa and received interventions. The only modality investigated in this group was intravenous injection of mesenchymal stem cells.

#### Intra venous injection of mesenchymal stem cells

The only study conducted to investigate regenerative medicine in the treatment of dystrophic epidermolysis bullosa focused on comparing intravenous injection of mesenchymal stem cells with a combination of intravenous injection of mesenchymal stem cells and cyclosporine suspension. The results indicated the significant effectiveness of both methods in decreasing the number of new blisters and promoting the healing rate of new blisters, with no significant difference observed between the two groups [17].

## Discussion

Regenerative medicine has emerged as a promising field in dermatology, offering innovative therapeutic approaches for various skin conditions. One of the key techniques utilized in regenerative medicine is platelet-rich plasma (PRP) injections, which have shown positive results in the treatment of conditions such as hair loss, pigment disorders, and inflammatory skin conditions. One potential way PRP aids in healing inflammatory skin lesions is by releasing a variety of growth factors, such as platelet-derived growth factor (PDGF) and TGF-β, from alpha platelet granules. This increase in TGF-β levels can then trigger negative feedback within the signaling pathways involved in inflammation [1].

In the realm of hair loss, particularly androgenetic alopecia (AGA), PRP injections have been extensively studied [5, 20, 22]. Research suggests that PRP injections, especially when administered using the double spin method, can lead to increased hair growth and density, particularly of terminal hairs [25]. Additionally, combining PRP with growth factors such as basic fibroblast growth factor or Concentrated Growth Factor (CGF) has shown enhanced efficacy in promoting hair regrowth [40, 43].

For pigmentary disorders like vitiligo, regenerative methods such as autologous epidermal melanocyte/keratinocyte cell transplantation have shown promising results [48–55]. Studies indicate that transplantation of these cells can effectively repigment the affected areas, with techniques like Erbium YAG laser or motorized dermabrasion enhancing the outcomes [45]. Moreover, the combination of different regenerative methods, such as melanocyte transplantation and hair follicle origin transplantation, has demonstrated significant improvements in repigmentation outcomes [52].

In other dermatologic conditions like melasma, regenerative approaches using PRP have yielded positive results. Studies have shown that PRP treatments can lead to significant improvements in skin pigmentation compared to traditional treatments like hydroquinone [12, 68]. Furthermore, the use of alternative sources of mesenchymal stem cells, such as umbilical cord-derived cells, has shown promising effects in treating melasma [69].

Notably, regenerative techniques like adipose-derived stem cell treatments and PRP injections have also shown potential benefits in addressing skin disorders like lichen sclerosis et atrophicus (LSA), inflammatory acne vulgaris, chronic telogen effluvium, erosive oral lichen planus, and alopecia areata. Studies have demonstrated the effectiveness of these methods in controlling symptoms, promoting hair growth, and improving patient satisfaction [11, 13, 14, 17, 67, 70].

Overall, regenerative medicine presents a wide array of therapeutic options for dermatologic conditions, with ongoing research focusing on optimizing treatment protocols and enhancing patient outcomes in the field of dermatology.

## Conclusion

Regenerative medicine, as a new branch of medical science, plays an effective role in treating degenerative skin diseases. While this treatment is effective, it has not been associated with serious side effects for patients. Our conclusion in this systematic review study was limited to primary skin diseases, and secondary conditions such as burn scars, acne scars, diabetic wounds, etc., were not investigated. One limitation of our study was the limited access to the complete texts of certain articles. Additionally, our focus was solely on RCTs in this research, and we excluded both case reports and case series from our analysis. It is recommended to conduct more clinical trial studies to explore further the role of regenerative medicine in treating skin diseases.

## Data Availability

The data that support the findings of this study are available from the corresponding author upon reasonable request.
